# Antimicrobial Multidrug Resistance and Mechanisms of Action: An Overview

**DOI:** 10.1155/bmri/8847267

**Published:** 2025-11-25

**Authors:** Abraham Belete Temesgen, Samuel Atalay Shiferaw

**Affiliations:** ^1^ Department of Veterinary Pathobiology, College of Veterinary Medicine and Animal Sciences, University of Gondar, Gondar, Ethiopia, uog.edu.et; ^2^ Amhara National Regional Health Bureau, Bahir Dar, Amhara, Ethiopia

**Keywords:** acquired resistance, antimicrobial multidrug resistance, bacteria, fungi, intrinsic resistance, parasites, viruses

## Abstract

Antimicrobial multidrug resistance is the ability of microorganisms to withstand the effects of several antimicrobial agents, presenting a major challenge to modern healthcare systems worldwide. Although considerable research has been conducted, the molecular and evolutionary mechanisms underlying resistance are still not completely understood. This review brings together current knowledge to explain how resistance originates, spreads, and persists in different pathogens. Microorganisms may show primary resistance, which arises naturally without prior exposure to drugs, or acquired resistance, which develops after contact with antimicrobial agents. Intrinsic resistance is related to structural or functional traits that are naturally present in specific species. Strains that are extensively resistant demonstrate survival against a wide range of important drugs, while clinical resistance becomes evident when standard treatments fail to control infections effectively. Pathogens employ several mechanisms, including enzymatic inactivation of drugs, modification of target sites, reduced drug uptake, and active efflux systems. Parasitic and fungal pathogens often rely on impaired drug transport and altered molecular targets, whereas viruses adopt multiple strategies to escape the activity of antiviral drugs. The appearance of highly resistant organisms such as methicillin‐resistant *Staphylococcus aureus* reflects the growing threat of so‐called superbugs. The rapid spread of resistance, driven by genetic mutations and horizontal gene transfer, highlights its ability to disseminate quickly within microbial populations. A clear understanding of these molecular processes is essential to guide the development of new therapeutic strategies, improve clinical management, and strengthen global efforts to control antimicrobial resistance.

## 1. Introduction

Antimicrobial multidrug resistance (MDR) has emerged as a critical and escalating global public health concern, characterized by the ability of certain microorganisms to withstand the effects of a broad spectrum of antimicrobial agents. This phenomenon undermines the efficacy of conventional infection control and treatment strategies, as consistently reported in the scientific literature [1–4]. The mechanisms that drive MDR are highly complex, involving diverse biochemical and physiological processes. These include alterations in drug targets that reduce binding affinity, increased activity of efflux pumps that expel antimicrobial agents from microbial cells, and enzymatic degradation or modification of compounds that neutralize their activity [5–7].

The interaction of these resistance mechanisms presents substantial challenges for both scientific understanding and clinical management. Despite considerable progress in antimicrobial research, knowledge of the molecular and ecological factors that contribute to the emergence and spread of resistance remains incomplete. This knowledge gap continues to hinder the development of effective preventive and therapeutic strategies, highlighting the urgent need for sustained research and innovation in the field [6, 8].

Antimicrobial agents are specialized compounds capable of inhibiting or killing microorganisms at very low concentrations, reflecting their potency and therapeutic value [9, 10]. Among them, antibiotics represent the most important class. Typically derived from the metabolic processes of microorganisms, antibiotics serve as an evolutionary strategy to eliminate microbial competition [11, 12]. These compounds may originate from natural sources, such as bacteria and fungi, or be produced synthetically. Synthetic antimicrobial agents are often developed from organic compounds or chemical dyes through sophisticated reactions, allowing the design of drugs with improved specificity and efficacy [9, 13, 14].

Since their introduction more than six decades ago, antibiotics have revolutionized medical practice by enabling the effective treatment of previously fatal or debilitating bacterial infections. Their widespread use has markedly reduced mortality and morbidity associated with infectious diseases, thereby transforming public health outcomes worldwide. These achievements are well documented in both clinical and epidemiological studies [15, 16]. However, the growing prevalence of antimicrobial resistance now threatens to undermine these gains. Pathogens responsible for serious diseases such as pneumonia, septicemia, gonorrhea, tuberculosis, and wound infections are increasingly showing resistance to multiple first‐line and last‐resort antibiotics [17–19].

Microorganisms display a remarkable ability to adapt, developing sophisticated mechanisms that allow them to evade antimicrobial action. This evolutionary resilience enables them to persist and proliferate even under therapeutic pressure, complicating infection management [20–22]. The widespread and often inappropriate use of antibiotics in human healthcare, veterinary medicine, and agriculture has further intensified this problem. Such practices impose selective pressure on microbial populations, encouraging the emergence and dissemination of resistant strains while reducing treatment effectiveness. The consequences are extensive, leading to prolonged illness, higher healthcare costs, and increased mortality. Tackling this threat requires a multifaceted and coordinated response, including rational use of antibiotics through antimicrobial stewardship programs, strengthening of surveillance systems, investment in the discovery of novel therapeutics, and the application of effective infection prevention and control measures [23, 24].

Antibiotic resistance remains one of the most pressing challenges in modern healthcare, posing significant risks to effective treatment and patient outcomes [6, 25, 26]. Although extensive research has been carried out, many aspects of the molecular mechanisms, evolutionary dynamics, and ecological factors underlying resistance remain poorly understood. This review is aimed at synthesizing current knowledge to improve understanding of antimicrobial resistance mechanisms and supporting the development of informed and effective interventions.

## 2. Antimicrobial MDR

Antimicrobial MDR represents a critical and increasingly urgent challenge in contemporary medicine. It is characterized by the ability of diverse microorganisms, including bacteria, viruses, fungi, and parasites, to develop resistance to a broad spectrum of antimicrobial agents. This resistance arises through genetic mutations and biochemical adaptations that enable pathogens to evade the pharmacological actions of drugs that were once effective [27]. The emergence and spread of MDR are influenced by multiple interrelated factors. Prominent among these are the widespread overuse and inappropriate prescribing of antibiotics, inadequate infection prevention and control practices, and the remarkable evolutionary capacity of microorganisms to adapt and persist under antimicrobial selective pressure [28, 29].

The clinical implications of antimicrobial resistance are profound, as it severely compromises the efficacy of existing treatment options. Infections that were once considered manageable have, in recent decades, become increasingly difficult to treat [30, 31]. This growing resistance undermines the protective and curative potential of antimicrobial agents, largely as a result of their irrational and excessive use in human medicine, veterinary medicine, and agriculture [30, 32, 33]. Several pathogens exemplify the severity of MDR. Notable among these are drug‐resistant *Mycobacterium tuberculosis*, methicillin‐resistant *Staphylococcus aureus* (MRSA), vancomycin‐resistant enterococcus (VRE), MDR *Neisseria gonorrhoeae*, and various MDR gram‐negative bacteria. These pathogens pose significant threats to global health by limiting therapeutic options and increasing the overall disease burden [30, 34–36].

### 2.1. Classification of MDR

The effective management of infectious diseases relies heavily on the administration of appropriate antimicrobial agents in accurate dosages and for the prescribed duration, ensuring the complete eradication of the causative pathogens. Inadequate dosing, irregular administration, or premature discontinuation of treatment often allows a portion of the microbial population to survive. These surviving organisms may subsequently acquire or enhance resistance mechanisms, ultimately leading to higher levels of drug resistance [37].

Clinical treatment failure, however, cannot be attributed solely to antimicrobial resistance. Host‐related factors also play a significant role. Impaired immune responses, reduced drug bioavailability, or accelerated metabolic clearance may limit the effectiveness of antimicrobial agents at the site of infection, thereby contributing to poor therapeutic outcomes. MDR is generally categorized into two main forms. Primary or intrinsic resistance refers to an inherent, genetically determined insensitivity of microorganisms to specific antimicrobials. Secondary or acquired resistance develops as a consequence of selective pressures following previous drug exposure or incomplete therapeutic interventions. Each form of resistance presents distinct clinical challenges and underscores the need to refine therapeutic strategies in order to manage drug‐resistant infections effectively [38, 39].

#### 2.1.1. Primary Resistance

Primary resistance refers to the phenomenon in which a microorganism demonstrates resistance to a specific antimicrobial agent even though the host has had no prior exposure to that drug. This indicates that resistance is already present before the initiation of treatment. A well‐documented example is primary drug‐resistant tuberculosis, in which individuals who have never received antitubercular therapy are infected with *Mycobacterium tuberculosis* strains resistant to one or more first‐line drugs [40–42]. In contrast to acquired resistance, which develops during or after therapeutic intervention, primary resistance reflects the transmission of resistant strains already circulating within the population. The emergence and persistence of primary resistance are strongly influenced by inadequate infection control measures, delays in diagnosis, inappropriate use of antimicrobial agents, and increased global travel that facilitates the international spread of resistant pathogens [43, 44].

#### 2.1.2. Secondary Resistance

Secondary resistance, also referred to as acquired resistance, describes the development of resistance in a microorganism following exposure to a specific antimicrobial agent. In contrast to primary resistance, where the pathogen is resistant before any treatment, secondary resistance arises in pathogens that were initially susceptible but later acquire the ability to evade the drug′s effects through adaptive mechanisms [45, 46]. This form of resistance commonly develops under selective pressure created by antimicrobial therapy, which favors the survival and multiplication of resistant mutants within the microbial population. A well‐recognized example is acquired drug‐resistant tuberculosis, in which *Mycobacterium tuberculosis* strains isolated from patients who are currently receiving or have previously undergone antitubercular therapy for at least 1 month demonstrate resistance to one or more first‐line drugs [47, 48].

##### 2.1.2.1. Intrinsic Resistance

Intrinsic resistance, also known as innate resistance or insensitivity, refers to the natural ability of certain microorganisms to withstand the effects of specific antimicrobial agents without prior exposure or genetic alteration. This resistance arises from structural and functional characteristics inherent to the organism that make it nonsusceptible to particular classes of drugs [49, 50]. The mechanisms underlying intrinsic resistance include the inability of the antimicrobial agent to bind effectively to its target site, restricted permeability of the microbial cell envelope, the activity of chromosomally encoded efflux pumps that expel the drug, and the endogenous production of enzymes capable of inactivating antimicrobial compounds [51, 52].

Well‐documented examples illustrate these principles. Gram‐positive bacteria are intrinsically resistant to aztreonam, a monobactam antibiotic, because the drug is ineffective against their cell wall structure. Gram‐negative bacteria demonstrate intrinsic resistance to vancomycin since the large molecular size of the drug prevents penetration through their outer membrane. Similarly, anaerobic bacteria are resistant to aminoglycosides because drug uptake requires oxygen, while aerobic bacteria display intrinsic resistance to metronidazole, which becomes active only under anaerobic conditions [53–56].

##### 2.1.2.2. Extensive Resistance

Extensive resistance, more precisely described as extensively drug‐resistant (XDR), represents one of the most advanced and concerning forms of antimicrobial resistance. It is defined as the ability of a microorganism to resist the effects of at least one agent in all but two or fewer categories of antimicrobial drugs, including some of the most potent agents currently available for clinical use. This form of resistance typically develops after the failure of first‐line therapies, particularly in patients with prolonged or inappropriate antimicrobial exposure. The emergence of XDR underscores the growing limitations of existing treatment regimens and the serious threat posed by the continuous evolution of resistance mechanisms [55].

A prominent example is extensively drug‐resistant tuberculosis (XDR‐TB), caused by strains of *Mycobacterium tuberculosis* that are resistant not only to isoniazid and rifampin, which are the cornerstone first‐line antitubercular drugs, but also to any fluoroquinolone and at least one of the second‐line injectable agents such as amikacin, kanamycin, or capreomycin. This resistance profile severely restricts therapeutic options and is commonly associated with poorer clinical outcomes, extended treatment durations, and higher mortality rates [57, 58].

#### 2.1.3. Clinical Resistance

**Figure 1:**
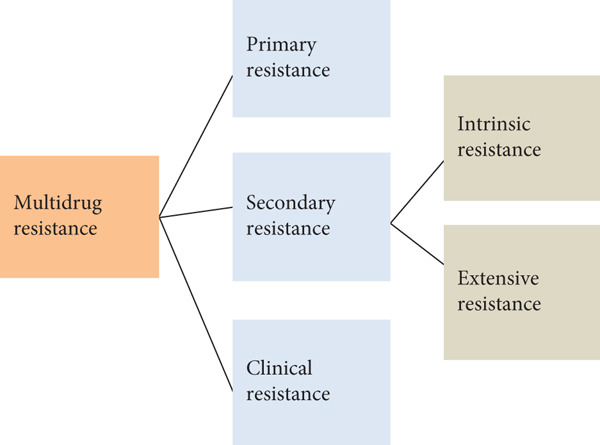
Classification of multidrug resistance (MDR) [38].

Clinical resistance (Figure 1) refers to the situation in which a microorganism causing an infection exhibits resistance to an antimicrobial agent at concentrations that exceed those achievable in the patient through standard therapeutic dosing. In such cases, the minimum inhibitory concentration (MIC) required to inhibit or eliminate the pathogen is substantially higher than the drug levels that can be safely attained in the patient′s bloodstream or tissues [59]. As a result, treatment failure, persistent or recurrent infections, and an increased risk of complications may occur.

Clinical resistance is distinct from microbiological resistance in that it emphasizes the ineffectiveness of a drug in real therapeutic contexts, even when some degree of in vitro activity is present. Managing infections caused by clinically resistant organisms often requires alternative strategies such as higher drug dosages, combination antimicrobial therapy, or the use of novel agents. These approaches, however, carry additional risks, including drug toxicity, adverse effects, and higher healthcare costs, which complicate treatment decisions and patient outcomes [60].

### 2.2. MDR Bacteria and Mode of Action

MDR bacteria (Table 1) have acquired the ability to withstand the effects of multiple commonly used antibiotics, representing a significant challenge in both clinical and environmental contexts (Table 1). These resistant strains are found not only in healthcare settings but also in natural environments such as water and soil, where they contribute to the ongoing dissemination of resistance genes [62, 63]. Although infections caused by MDR bacteria may present with clinical manifestations similar to those caused by susceptible strains, treatment is considerably more difficult due to the limited availability of effective therapeutic options. The complexity of managing MDR infections is further compounded by resistance not only to first‐line antibiotics but also to multiple classes of second‐line drugs. Prominent MDR pathogens include *Pseudomonas aeruginosa*, *Staphylococcus aureus* (particularly MRSA), *Escherichia coli*, *Acinetobacter baumannii*, *Klebsiella pneumoniae*, *Mycobacterium tuberculosis*, and *Neisseria gonorrhoeae* [59, 64].

**Table 1:** Common multidrug‐resistant bacteria and the diseases they cause.

**Bacteria**	**Resistant to**	**Diseases**	**References**
*Escherichia coli*	Cephalosporins and fluoroquinolones	Urinary tract infections and bloodstream infections	[6]
*Klebsiella pneumoniae*	Cephalosporins and carbapenems	Pneumonia, bloodstream infections, and urinary tract infections	[38]
*Staphylococcus aureus*	Methicillin	Wound infections and bloodstream infections	[61]
*Streptococcus pneumoniae*	Penicillin	Pneumonia, meningitis, and otitis	[27]
Nontyphoidal *Salmonella*	Fluoroquinolones	Foodborne diarrhea and bloodstream infections	[38]
*Shigella* species	Fluoroquinolones	Diarrhea (bacillary dysentery)	[38]
*Neisseria gonorrhoeae*	Cephalosporins	Gonorrhea	[6]
*Mycobacterium tuberculosis*	Rifampicin, isoniazid, and fluoroquinolones	Tuberculosis	[27]

Antibiotic resistance in bacteria arises through a variety of sophisticated mechanisms that enable pathogens to evade antimicrobial action. One key mechanism is drug inactivation or modification, in which bacteria produce enzymes such as *β*‐lactamases that degrade or alter antibiotics, including Penicillin G. These enzymes chemically modify the drug through processes such as acetylation or phosphorylation, thereby impairing its ability to bind bacterial targets and inhibit essential functions such as protein synthesis [65–67]. Another important mechanism involves the modification of drug target sites. Changes in penicillin‐binding proteins or ribosomal protection proteins reduce antibiotic binding affinity and prevent effective inhibition of bacterial processes [68–70]. Bacteria may also develop altered metabolic pathways; for instance, the modification or absence of para‐aminobenzoic acid, a precursor required for folic acid and nucleic acid synthesis, allows bacteria to bypass the inhibitory effects of drugs targeting these pathways [68, 70]. Finally, reduced intracellular drug accumulation occurs either through decreased permeability of the bacterial cell membrane or through enhanced drug efflux systems, which actively pump antibiotics out of the cell, lowering intracellular drug concentrations below therapeutic thresholds [66, 71, 72].

### 2.3. MDR Protozoa and Helminths and Mode of Action

Parasitic protozoa (Table 2) are responsible for numerous significant diseases in both humans and animals, despite being eukaryotic organisms with cellular organelles and metabolic pathways similar to those of their hosts [73]. These protozoa have evolved diverse strategies to survive and evade the effects of antimicrobial agents. For example, *Plasmodium* species, the causative agents of malaria, which are characterized by recurrent fever and anemia, have developed resistance to several frontline antimalarial drugs. Similarly, *Leishmania* species cause leishmaniasis, which can manifest in cutaneous, mucocutaneous, or visceral forms. *Trypanosoma brucei gambiense* and *Trypanosoma brucei rhodesiense* are responsible for African trypanosomiasis (sleeping sickness), with drug resistance complicating treatment. Additionally, *Toxoplasma gondii*, the agent of toxoplasmosis, poses significant risks, particularly to immunocompromised individuals and pregnant women [74–76].

**Table 2:** Common multidrug‐resistant parasites and the diseases they cause.

**Parasite**	**Resistant to**	**Diseases**	**References**
*Plasmodia* spp.	Chloroquine, artemisinin, and atovaquone	Malaria	[38]
*Leishmania* spp.	Pentavalent antimonials, miltefosine, paromomycin, and Amphotericin B	Leishmaniasis	[38]
*Schistosomes*	Praziquantel and oxamniquine	Schistosomiasis	[61]
*Entamoeba*	Metronidazole	Amoebiasis	[38]
*Trichomonas vaginalis*	Nitroimidazoles	Trichomoniasis	[27]
*Toxoplasma gondii*	Artemisinin, atovaquone, and sulfadiazine	Toxoplasmosis	[6]

Protozoal drug resistance is mediated by several sophisticated mechanisms. One primary mechanism is reduced drug uptake, often resulting from the loss or mutation of transporters required for drug entry. This mechanism is well documented in African trypanosomes, where resistance to arsenicals and diamidines is associated with the absence of specific transporters [77, 78]. Protozoa also employ active drug efflux via transport proteins such as P‐glycoproteins and ATPases, which extrude drugs including emetine, mefloquine, and antimonials, thereby lowering intracellular drug concentrations and reducing therapeutic efficacy [79, 80]. Alterations in drug targets also contribute to resistance, as exemplified by mutations in the dihydrofolate reductase–thymidylate synthase enzyme in *Plasmodium* species, which reduce the binding affinity of antifolate drugs [81]. Loss of drug activation is another important mechanism. For instance, in *Trichomonas* and *Giardia*, decreased levels of the electron donor ferredoxin impair metronidazole activation, rendering the drug less effective [82–87].

Helminths, including nematodes, cestodes, and trematodes, also represent a major global health burden in humans and animals [88–90]. Resistance in helminths frequently arises from genetic alterations affecting drug targets. Single‐nucleotide polymorphisms (SNPs) can modify the structure or function of proteins targeted by anthelmintics, reducing drug binding and efficacy. These mutations may arise spontaneously or be induced by selective drug pressure, complicating control measures [91, 92].

Alterations in drug transport similarly contribute to helminth resistance. P‐glycoprotein, a membrane efflux pump studied extensively in cancer cells, also functions in helminths to extrude therapeutic agents. This mechanism reduces intracellular concentrations of several anthelmintics, including ivermectin, benzimidazoles, and imidazothiazole derivatives, thereby diminishing drug effectiveness [93, 94].

Enhanced drug metabolism further contributes to resistance in both protozoa and helminths. Increased metabolic activity allows parasites to detoxify or inactivate drugs before they exert cytotoxic effects. For example, anthelmintics such as praziquantel and artemisinin, used against *Schistosoma* species, generate free radicals to damage parasite cells. Resistant parasites often exhibit an increased capacity to neutralize these radicals, reducing the efficacy of treatment [95–98].

### 2.4. MDR in Viruses and Modes of Action

Viruses (Table 3) have evolved diverse mechanisms that allow them to resist antiviral drugs, resulting in the emergence of viral strains resistant to nearly all currently available treatments [100, 101]. Although viral resistance is not a new phenomenon, its prevalence and complexity have increased considerably over time. The remarkable adaptability of viruses, driven by rapid genetic mutation and replication, enables them to alter drug targets, modify replication pathways, and, in some cases, enhance drug efflux, thereby complicating the management of viral infections. A comprehensive understanding of these resistance mechanisms is essential for the development of effective antiviral therapies and for controlling resistant viral strains [101, 102].

**Table 3:** Common multidrug‐resistant viruses and the diseases they cause.

**Virus**	**Resistant to**	**Diseases**	**References**
Cytomegalovirus (CMV)	Ganciclovir and foscarnet	In AIDS and oncology patients	[99]
Herpes simplex virus (HSV)	Acyclovir, famciclovir, and valacyclovir	Herpes simplex	[6]
Human immunodeficiency virus (HIV)	Antiretroviral drugs	AIDS	[38]
Influenza virus	Adamantane derivatives (amantadine and rimantadine) and neuraminidase inhibitors	Influenza	[27]
Varicella zoster virus	Acyclovir and valacyclovir	Chicken pox	[6]
Hepatitis B virus (HBV)	Lamivudine	Hepatitis B	[38]

Many antiviral agents target viral enzymes, such as DNA polymerase or the reverse transcriptase activity critical for viral replication. Resistance frequently arises from mutations within the reverse transcriptase domain of the viral polymerase gene. These mutations induce structural changes in the enzyme, reducing drug binding affinity or allowing the enzyme to maintain function despite inhibitor presence. For example, conformational alterations in the enzyme′s active site may prevent effective drug interaction, enabling viral persistence even during treatment. These sophisticated molecular adaptations underscore the need for continuous research to elucidate viral resistance mechanisms and to identify novel therapeutic strategies capable of overcoming antiviral resistance [99, 103].

### 2.5. MDR in Fungi and Modes of Action

Fungal cells (Table 4) have developed a range of sophisticated mechanisms to resist antifungal agents, which significantly complicates therapeutic interventions. One common resistance strategy is the modification of drug targets, while a more frequent mechanism involves the upregulation of drug efflux pumps. These pumps actively expel antifungal compounds before they reach their intracellular targets, thereby reducing drug efficacy. The fungal cell wall also plays a crucial role in modulating susceptibility to antifungal drugs [105, 106]. For example, antifungal agents that inhibit ergosterol biosynthesis, such as polyenes, disrupt fungal growth by depleting ergosterol in the plasma membrane. This depletion reduces membrane permeability and impairs drug uptake [107, 108]. Changes in membrane components, including *β*‐1,3‐glucan and lipid composition, can eliminate or modify binding sites for antifungals such as echinocandins, preventing effective drug–target interaction and conferring resistance [109, 110].

**Table 4:** Common multidrug‐resistant fungi and the diseases they cause.

**Fungus**	**Resistant to**	**Diseases**	**References**
*Candida* spp.	Fluconazole and echinocandins	Candidiasis	[38]
*Cryptococcus neoformans*	Fluconazole	Cryptococcosis	[104]
*Aspergillus* spp.	Azoles	Aspergillosis	[27]
*Scopulariopsis* spp.	Amphotericin B, flucytosine, and azoles	Onychomycosis	[104]

Mutations in genes encoding essential drug targets result in molecular alterations that allow fungi to maintain vital cellular functions despite antifungal pressure, reducing susceptibility [107, 111]. Overexpression of drug target enzymes is another important mechanism, enabling fungi to circumvent inhibitory effects by modifying metabolic pathways. This adaptation is particularly evident in resistance to azoles and allylamines, where alternative biosynthetic pathways or changes in protein synthesis sustain fungal viability despite drug exposure [112–114]. Candida species frequently develop resistance to azoles after prolonged treatment, often necessitating a switch to alternative antifungal classes. In addition, infections caused by MDR fungi such as *Scedosporium prolificans* present significant clinical challenges due to resistance to multiple antifungal agents and high associated mortality [115–119].

### 2.6. Mechanisms and the Emergence of Antibiotic Resistance

The emergence of so‐called superbugs was notably observed in the summer of 2002 when *Staphylococcus aureus*, a common but potentially lethal bacterium, acquired a novel antibiotic resistance gene. This strain, isolated from foot ulcers in diabetic patients in Detroit, Michigan, was identified as MRSA, a pathogen already recognized as a serious challenge within healthcare settings [120–123]. The situation escalated with the discovery of a variant resistant to vancomycin, previously considered a last‐resort antibiotic effective against *S. aureus*. This vancomycin‐resistant *S. aureus* (VRSA) strain exhibited resistance to multiple antibiotics, including ciprofloxacin, methicillin, and penicillin. Concurrently, VREs were isolated from the same patient, further complicating the management of these MDR infections [124, 125].

Genetic analyses revealed a critical development. The initially vancomycin‐sensitive *S. aureus* acquired the vancomycin resistance gene VanA from VRE through horizontal gene transfer (HGT) via conjugation. This process, in which genetic material is exchanged directly between bacteria, posed a significant public health threat by enabling *S. aureus*, previously susceptible to one of the few remaining effective antibiotics, to resist vancomycin [120, 122, 126]. The integration of the VanA gene, known for conferring high‐level vancomycin resistance, represented a dramatic escalation in the challenge of treating *S. aureus* infections. This case highlights the alarming potential for genetic exchange among bacterial populations to rapidly amplify antimicrobial resistance, undermining current treatment strategies [120, 122, 126].

Bacteria employ a diverse array of mechanisms to resist antimicrobial agents, with strategies varying widely across species and drug classes, reflecting the complexity of antimicrobial resistance [122, 127]. Susceptible bacteria may develop resistance through spontaneous genetic mutations or acquire resistance genes from other bacteria. These processes involve multiple biochemical and genetic mechanisms, including mutation‐induced alterations in bacterial proteins or enzymes that reduce drug susceptibility, enzymatic degradation of antibiotics such as *β*‐lactamases that inactivate *β*‐lactam drugs, and the use of efflux pumps that actively expel antimicrobial compounds, lowering their intracellular concentrations. In addition, HGT mechanisms, including conjugation, transformation, and transduction, facilitate the dissemination of resistance genes between bacteria, promoting the rapid spread of resistance traits across microbial populations [128–131].

#### 2.6.1. Mutation

Mutation refers to changes in the DNA sequence of an organism that can lead to alterations in gene products, which are often the direct targets of antimicrobial agents. For example, fluoroquinolones exert their antibacterial effects by targeting bacterial enzymes such as DNA gyrase, which plays a vital role in DNA replication by introducing negative supercoils necessary for maintaining DNA structure and facilitating replication. Fluoroquinolones bind to DNA gyrase, inhibiting its activity and thereby preventing bacterial DNA replication, ultimately leading to cell death. Mutations in the genes encoding DNA gyrase can modify the enzyme′s structure, reducing the binding affinity of fluoroquinolones and conferring resistance. As a result, despite the presence of the drug, the mutated enzyme remains functional, allowing the bacterium to survive and proliferate even at therapeutic drug concentrations [132, 133].

Mutations within specific regions of genes encoding essential bacterial enzymes can diminish the efficiency of antimicrobial binding, enabling bacteria to maintain critical processes such as DNA replication in the presence of these drugs. Fluoroquinolones target both DNA gyrase and Topoisomerase IV, enzymes essential for bacterial DNA replication, and mutations in these enzymes can alter their conformation, thereby reducing drug binding and bactericidal activity. In addition, certain pathogens develop resistance by preventing drug entry into the bacterial cell. Many gram‐negative bacteria resist Penicillin G due to the inability of the drug to penetrate their outer membrane, which serves as a permeability barrier. Mutations in penicillin‐binding proteins, the targets of *β*‐lactam antibiotics including penicillin, also contribute to resistance by lowering the drug′s binding affinity. Similarly, decreased permeability of the bacterial cell wall can result in resistance to sulfonamides by restricting drug uptake and preventing the inhibition of folic acid synthesis [134–136].

#### 2.6.2. Destruction or Inactivation

Many bacteria have evolved genes encoding enzymes that chemically modify or degrade antimicrobial agents, effectively neutralizing their activity and rendering these drugs ineffective. A well‐known example of this resistance mechanism is the hydrolysis of the *β*‐lactam ring in penicillins by *β*‐lactamase enzymes, such as penicillinase. This enzymatic cleavage disrupts the *β*‐lactam ring, a critical structural component of the antibiotic, thereby inactivating the drug and preventing it from exerting bactericidal effects [134, 137]. Similarly, chloramphenicol, which contains hydroxyl groups, is inactivated by chloramphenicol acetyltransferase. This enzyme transfers acetyl groups from acetyl‐CoA to the drug molecule, neutralizing its antibacterial activity. Aminoglycoside antibiotics are also susceptible to enzymatic modification, which impairs their function. Acetyltransferases catalyze the addition of acetyl groups to amino groups of aminoglycosides, while other modifying enzymes, including phosphotransferases and adenyltransferases, add phosphate and adenyl groups, respectively, further altering the chemical structure of the drug and reducing its efficacy [134, 138].

#### 2.6.3. Efflux Pump

**Figure 2:**
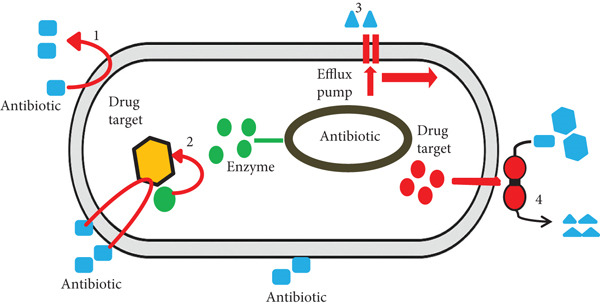
Mechanisms of antimicrobial multidrug resistance (MDR): (1) altering membrane permeability or using efflux pumps to prevent drug entry, (2) enzymatically modifying or degrading drugs, (3) altering drug targets to reduce susceptibility, and (4) modifying cellular targets to evade antimicrobial effects [28].

One of the most prevalent mechanisms of bacterial drug resistance is the active efflux of antimicrobial agents from the cell (Figure 2). This strategy involves energy‐dependent efflux pumps that transport drugs out of bacterial cells, lowering intracellular concentrations of antimicrobial agents to subinhibitory levels. By reducing the amount of drug that reaches its target site, these pumps effectively diminish the therapeutic efficacy of antimicrobial treatments. The energy required for this process is typically derived from cellular metabolism, allowing the pumps to expel a wide range of drugs across the bacterial membrane. Consequently, efflux pump activity significantly compromises antibiotic effectiveness and complicates the achievement of inhibitory or bactericidal drug concentrations within the cell [139–141].

Efflux pumps are complex transport systems that actively export antimicrobial compounds and other substances from bacterial cells, contributing substantially to MDR. Drugs that enter the bacterial cytoplasm via porin channels are expelled using energy‐dependent mechanisms, and the ability of these pumps to recognize and remove structurally diverse antibiotics has earned them the designation of MDR pumps. Two main categories of active efflux pumps exist: primary active transporters, which use ATP hydrolysis to power drug expulsion, and secondary active transporters, which exploit ion gradients to drive efflux. This dual mechanism enables bacteria to maintain low intracellular drug concentrations, effectively undermining antimicrobial activity [134, 142, 143].

Among primary active transporters, ATP‐binding cassette (ABC) transporters, also known as P‐glycoprotein transporters, play a crucial role in bacterial resistance by harnessing ATP hydrolysis to expel a broad spectrum of antimicrobial agents. Pathogenic bacteria, such as *Escherichia coli*, *Pseudomonas aeruginosa*, and *Staphylococcus aureus*, utilize these ABC transport systems to resist multiple classes of antibiotics. By actively removing drugs before they reach intracellular targets, these transporters markedly reduce treatment efficacy, posing significant challenges to infection control and therapeutic interventions [122].

#### 2.6.4. Measures to Reverse Resistance

HGT is a key genetic mechanism that strongly influences antibiotic resistance among microorganisms. This process enhances microbial survival against antimicrobial treatments and affects pathogen–host interactions and transmission patterns. HGT can impose fitness costs on microbes, such as reduced growth rates, diminished virulence, and lower transmission efficiency within hosts and in environmental reservoirs [23, 144–146]. Despite these costs, resistance determinants often persist and spread widely, highlighting the importance of strict infection control measures. Among these, consistent hand hygiene following patient contact is a fundamental but often underutilized practice essential for limiting the spread of resistant pathogens [147, 148].

Epidemiological studies of antibiotic resistance, such as erythromycin resistance in *Streptococcus pyogenes* and penicillin resistance in *Streptococcus pneumoniae*, demonstrate the adaptability and resilience of resistance in community settings. These findings underline the ongoing challenge of containing resistant infections and the need for continuous surveillance and management strategies [149–151].

Research has identified mechanisms that contribute to the long‐term maintenance of resistance in bacterial populations. One key process is compensatory evolution, in which bacteria acquire secondary mutations that reduce the fitness costs associated with resistance. This adaptation allows resistant strains to remain prevalent despite initial disadvantages, stabilizing resistance within microbial communities. While many resistance‐conferring mutations incur fitness penalties, such as slower growth or reduced pathogenicity, some mutations are effectively cost‐free. These low‐cost mutations promote the persistence and spread of resistance by lowering selective pressure for reversion to susceptibility, complicating efforts to control antimicrobial resistance at the population level [152, 153].

Public health interventions play an essential role in reducing resistance development. Washing raw fruits and vegetables removes resistant bacteria and antibiotic residues, decreasing the risk of introducing resistant organisms into the human microbiome. Completing prescribed antibiotic regimens is equally critical to ensure full pathogen eradication and prevent the survival of resistant subpopulations. Patients are advised against keeping leftover antibiotics for future use because this practice encourages inappropriate dosing and the emergence of resistance [154, 155]. While topical antibiotic ointments can help treat minor wounds, caution is advised regarding personal care products containing antibacterial agents. Some of these chemicals may unintentionally select for bacteria resistant to both the agents in the products and conventional antibiotics, potentially worsening resistance problems [156].

## 3. Conclusion

Antimicrobial MDR represents a critical and growing threat to global health. Its emergence is driven by natural evolutionary processes, yet its rapid spread is amplified by the inappropriate use of antimicrobial agents. Understanding the molecular mechanisms of resistance and the actions of antimicrobial drugs is essential for developing effective interventions and preserving the efficacy of current therapies. Combating this challenge requires coordinated efforts, including responsible antimicrobial stewardship, systematic surveillance, and ongoing investment in new drug development. Collaboration among researchers, clinicians, and public health authorities is vital to limit the spread of resistant pathogens, optimize treatment outcomes, and safeguard public health. Strengthening these integrated strategies will be essential to ensure that antimicrobial therapies remain effective for current and future populations.

## Ethics Statement

No human subjects were involved.

## Consent

The authors have nothing to report.

## Disclosure

All authors approved the final manuscript.

## Conflicts of Interest

The authors declare no conflicts of interest.

## Author Contributions

All authors contributed equally to the study.

## Funding

No funding was received for this manuscript.

## Data Availability

The authors have nothing to report.

## References

[1] Magiorakos A. P. , Srinivasan A. , Carey R. B. , Carmeli Y. , Falagas M. E. , Giske C. G. , Harbarth S. , Hindler J. F. , Kahlmeter G. , Olsson-Liljequist B. , Paterson D. L. , Rice L. B. , Stelling J. , Struelens M. J. , Vatopoulos A. , Weber J. T. , and Monnet D. L. , Multidrug-Resistant, Extensively Drug-Resistant and Pandrug-Resistant Bacteria: An International Expert Proposal for Interim Standard Definitions for Acquired Resistance, Clinical Microbiology and Infection. (2012) 18, no. 3, 268–281, 10.1111/j.1469-0691.2011.03570.x, 2-s2.0-84857646835, 21793988. 21793988

[2] World Health Organization (WHO) , Antimicrobial Resistance, 2018, https://www.who.int/news-room/fact-sheets/detail/antimicrobial-resistance.

[3] Abushaheen M. A. , Fatani A. J. , Alosaimi M. , Mansy W. , George M. , Acharya S. , Rathod S. , Divakar D. D. , Jhugroo C. , Vellappally S. , and Khan A. A. , Antimicrobial Resistance, Mechanisms and Its Clinical Significance, Disease-a-Month. (2020) 66, no. 6, 100971, 10.1016/j.disamonth.2020.100971. 32201008

[4] Scoffone V. C. , Barbieri G. , Irudal S. , Trespidi G. , and Buroni S. , New Antimicrobial Strategies to Treat Multi-Drug Resistant Infections Caused by Gram-Negatives in Cystic Fibrosis, Antibiotics. (2024) 13, no. 1, 10.3390/antibiotics13010071, 38247630. PMC10812592 38247630

[5] Liu B. and Pop M. , ARDB—Antibiotic Resistance Genes Database, Nucleic Acids Research. (2009) 37, no. supplement 1, D443–D447, 10.1093/nar/gkn656, 2-s2.0-58149183697, 18832362. 18832362 PMC2686595

[6] World Health Organization (WHO) , Antimicrobial Resistance: Global Report on Surveillance, 2014, https://www.who.int/publications/i/item/9789241564748.

[7] Dandachi I. , Chaddad A. , Hanna J. , Matta J. , and Daoud Z. , Understanding the Epidemiology of Multi-Drug Resistant Gram-Negative Bacilli in the Middle East Using a One Health Approach, Frontiers in Microbiology. (2019) 10, 10.3389/fmicb.2019.01941, 2-s2.0-85071944455, 31507558. PMC6716069 31507558

[8] Krajewska J. and Laudy A. E. , The European Medicines Agency Approved the New Antibacterial Drugs–Response to the 2017 WHO Report on the Global Problem of Multi-Drug Resistance, Advancements of Microbiology. (2021) 60, no. 4, 249–264, 10.21307/pm-2021.60.4.20.

[9] Laxminarayan R. , Duse A. , Wattal C. , Zaidi A. K. , Wertheim H. F. , Sumpradit N. , Vlieghe E. , Hara G. L. , Gould I. M. , Goossens H. , Greko C. , So A. D. , Bigdeli M. , Tomson G. , Woodhouse W. , Ombaka E. , Peralta A. Q. , Qamar F. N. , Mir F. , Kariuki S. , Bhutta Z. A. , Coates A. , Bergstrom R. , Wright G. D. , Brown E. D. , and Cars O. , Antibiotic Resistance—The Need for Global Solutions, Lancet Infectious Diseases. (2013) 13, no. 12, 1057–1098, 10.1016/S1473-3099(13)70318-9, 2-s2.0-84887627398, 24252483. 24252483

[10] Assefa M. , Multi-Drug Resistant Gram-Negative Bacterial Pneumonia: Etiology, Risk Factors, and Drug Resistance Patterns, Pneumonia. (2022) 14, no. 1, 10.1186/s41479-022-00096-z, 35509063. PMC9069761 35509063

[11] Talaro K. P. , Talaro A. , Delisle G. , and Tomalty L. , Foundations in Microbiology, 1996, Wm. C. Brown.

[12] Chauhan A. , Kumar M. , Kumar A. , and Kanchan K. , Comprehensive Review on Mechanism of Action, Resistance and Evolution of Antimycobacterial Drugs, Life Sciences. (2021) 274, 119301, 10.1016/j.lfs.2021.119301, 33675895. 33675895

[13] Bonhi K. L. R. and Imran S. , Role of Bacteriocin in Tackling the Global Problem of Multi-Drug Resistance: An Updated Review, Bioscience Biotechnology Research Communications. (2019) 12, no. 3, 601–608, 10.21786/bbrc/12.3/8.

[14] Sharma N. , Chhillar A. K. , Dahiya S. , Choudhary P. , Punia A. , and Gulia P. , Antibiotic Adjuvants: A Promising Approach to Combat Multidrug Resistant Bacteria, Current Drug Targets. (2021) 22, no. 12, 1334–1345, 10.2174/1389450122666210120084406. 33494671

[15] Laxminarayan R. and Heymann D. L. , Challenges of Drug Resistance in the Developing World, BMJ. (2012) 344, no. apr03 2, e1567, 10.1136/bmj.e1567, 2-s2.0-84859712185. 22491075

[16] Adeeyo A. O. , Edokpayi J. N. , Alabi M. A. , Msagati T. A. , and Odiyo J. O. , Plant Active Products and Emerging Interventions in Water Potabilisation: Disinfection and Multi-Drug Resistant Pathogen Treatment, Clinical Phytoscience. (2021) 7, no. 1, 10.1186/s40816-021-00258-4.

[17] World Health Organization (WHO) , The Evolving Threat of Antimicrobial Resistance: Options for Action, 2013, https://iris.who.int/handle/10665/44812.

[18] Shaikh S. , Nazam N. , Rizvi S. M. D. , Ahmad K. , Baig M. H. , Lee E. J. , and Choi I. , Mechanistic Insights Into the Antimicrobial Actions of Metallic Nanoparticles and Their Implications for Multidrug Resistance, International Journal of Molecular Sciences. (2019) 20, no. 10, 10.3390/ijms20102468, 2-s2.0-85066429640, 31109079. PMC6566786 31109079

[19] Ashraf S. , Bashir N. , Rashid N. , Chughtai A. H. , Zia K. M. , Majeed S. , Ashiq M. N. , Murtaza G. , and Najam-ul-Haq M. , Introduction to Drugs, Drug Targets and Drug Resistance, Biochemistry of Drug Resistance, 2021, Springer International Publishing, Cham, 1–31, 10.1007/978-3-030-76320-6_1.

[20] Boucher H. W. , Talbot G. H. , Benjamin D. K.Jr., Bradley J. , Guidos R. J. , Jones R. N. , Murray B. E. , Bonomo R. A. , Gilbert D. , and the Infectious Diseases Society of America , 10 x ′20 Progress--Development of New Drugs Active Against Gram-Negative Bacilli: An Update From the Infectious Diseases Society of America, Clinical Infectious Diseases. (2013) 56, no. 12, 1685–1694, 10.1093/cid/cit152, 2-s2.0-84878278251, 23599308. 23599308 PMC3707426

[21] Simons A. , Alhanout K. , and Duval R. E. , Bacteriocins, Antimicrobial Peptides From Bacterial Origin: Overview of Their Biology and Their Impact Against Multidrug-Resistant Bacteria, Microorganisms. (2020) 8, no. 5, 10.3390/microorganisms8050639, 32349409. PMC7285073 32349409

[22] Ali S. , Sharma B. , and Rawat A. , Bacterial Cell Wall Nature and Its Mode of Resistance Against Antibiotic Drugs: An Overview, Journal of Mountain Research. (2021) 16, no. 3, 387–395, 10.51220/jmr.v16i3.38.

[23] Todar K. , Todar’s Online Textbook of Bacteriology, 2006, University of Wisconsin–Madison Department of Bacteriology, 1–580.

[24] Abdulla-Eltawaty S. I. , Busba A. O. , Omare M. E. A. , Almagboul A. Z. , Yagoub S. O. , Alramli A. , Ahmed A. , and Abdalbagi-Dafalla M. , Antimicrobial Activity of Libyan *Salvia fruticosa* Mil and Multi-Drug Resistant Bacteria, European Journal of Pharmaceutical and Medical Research. (2021) 8, no. 1, 416–423.

[25] Chinemerem Nwobodo D. , Ugwu M. C. , Oliseloke Anie C. , Al-Ouqaili M. T. , Chinedu Ikem J. , Victor Chigozie U. , and Saki M. , Antibiotic Resistance: The Challenges and Some Emerging Strategies for Tackling a Global Menace, Journal of Clinical Laboratory Analysis. (2022) 36, no. 9, e24655, 10.1002/jcla.24655, 35949048. 35949048 PMC9459344

[26] Alara J. A. and Alara O. R. , An Overview of the Global Alarming Increase of Multiple Drug Resistant: A Major Challenge in Clinical Diagnosis, Infectious Disorders–Drug Targets. (2024) 24, no. 3, 26–42, 10.2174/1871526523666230725103902. 37909431

[27] Huttner A. , Harbarth S. , Carlet J. , Cosgrove S. , Goossens H. , Holmes A. , Jarlier V. , Voss A. , Pittet D. , and the World Healthcare-Associated Infections Forum participants , Antimicrobial Resistance: A Global View From the 2013 World Healthcare-Associated Infections Forum, Antimicrobial Resistance and Infection Control. (2013) 2, no. 1, 10.1186/2047-2994-2-31, 2-s2.0-84903631541, 24237856. PMC4131211 24237856

[28] Roca I. , Akova M. , Baquero F. , Carlet J. , Cavaleri M. , Coenen S. , Cohen J. , Findlay D. , Gyssens I. , de la Heure O. E. , Kahlmeter G. , Kruse H. , Laxminarayan R. , Liébana E. , López-Cerero L. , MacGowan A. , Martins M. , Rodríguez-Baño J. , Rolain J. M. , Segovia C. , Sigauque B. , Tacconelli E. , Wellington E. , and Vila J. , The Global Threat of Antimicrobial Resistance: Science for Intervention, New Microbes and New Infections. (2015) 6, 22–29, 10.1016/j.nmni.2015.02.007, 2-s2.0-84930202457, 26029375. 26029375 PMC4446399

[29] Goyal B. , Verma N. , Kharewal T. , Gahlaut A. , and Hooda V. , Structural Effects of Nanoparticles on Their Antibacterial Activity Against Multi-Drug Resistance, Inorganic and Nano-Metal Chemistry. (2024) 54, no. 6, 534–546, 10.1080/24701556.2021.2025103.

[30] National Institute of Allergy and Infectious Diseases (NIAID) , Antibiotic and Antimicrobial Resistance, 2009, https://www.niaid.nih.gov/research/antimicrobial-resistance.

[31] Dolma K. G. and WM M. D. , *Acinetobacter baumannii*: An Overview of Emerging Multidrug-Resistant Pathogen, Medical Journal of Malaysia. (2022) 77, no. 3. 35638493

[32] Saha M. and Sarkar A. , Review on Multiple Facets of Drug Resistance: A Rising Challenge in the 21st Century, Journal of Xenobiotics. (2021) 11, no. 4, 197–214, 10.3390/jox11040013, 34940513. 34940513 PMC8708150

[33] Duan C. , Yu M. , Xu J. , Li B. Y. , Zhao Y. , and Kankala R. K. , Overcoming Cancer Multi-Drug Resistance (MDR): Reasons, Mechanisms, Nanotherapeutic Solutions, and Challenges, Biomedicine & Pharmacotherapy. (2023) 162, 114643, 10.1016/j.biopha.2023.114643, 37031496. 37031496

[34] Johnson S. R. and Morse S. A. , Antibiotic Resistance in *Neisseria gonorrhoeae*: Genetics and Mechanisms of Resistance, Sexually Transmitted Diseases. (1988) 15, no. 4, 217–224, 10.1097/00007435-198810000-00008, 2-s2.0-0024209705. 3147526

[35] Swamy M. K. , Purushotham B. , and Sinniah U. R. , Camptothecin: Occurrence, Chemistry and Mode of Action, Bioactive Natural Products for Pharmaceutical Applications, 2021, Springer International Publishing, Cham, 311–327, 10.1007/978-3-030-54027-2_9.

[36] Singh V. and Chibale K. , Strategies to Combat Multi-Drug Resistance in Tuberculosis, Accounts of Chemical Research. (2021) 54, no. 10, 2361–2376, 10.1021/acs.accounts.0c00878, 33886255. 33886255 PMC8154215

[37] Giurazza R. , Mazza M. C. , Andini R. , Sansone P. , Pace M. C. , and Durante-Mangoni E. , Emerging Treatment Options for Multi-Drug-Resistant Bacterial Infections, Life. (2021) 11, no. 6, 10.3390/life11060519, 34204961. PMC8229628 34204961

[38] Tanwar J. , Das S. , Fatima Z. , and Hameed S. , Multidrug Resistance: An Emerging Crisis, Interdisciplinary Perspectives on Infectious Diseases. (2014) 2014, 541340, 10.1155/2014/541340, 2-s2.0-84925862336. 25140175 PMC4124702

[39] Harikumar G. and Krishanan K. , The Growing Menace of Drug Resistant Pathogens and Recent Strategies to Overcome Drug Resistance: A Review, Journal of King Saud University–Science. (2022) 34, no. 4, 101979, 10.1016/j.jksus.2022.101979.

[40] Paramasivan C. N. , Bhaskaran K. , Venkataraman P. , Chandrasekaran V. , and Narayanan P. R. , Surveillance of Drug Resistance in Tuberculosis in the State of Tamil Nadu, Indian Journal of Tuberculosis. (2000) 47, no. 1, 27–33.

[41] Vaidya S. , Muley S. , Kulkarni M. , and Koppikar G. , To Study Incidence of Multi Drug Resistant Tuberculosis in Mumbai, LIFE: International Journal of Health and Life-Sciences. (2015) 1, no. 1, 122–138, 10.20319/lijshls.2015.s11.122138.

[42] Sukumaran D. P. and Abdulla M. H. , Can Bio-Nanotechnology Be Effective Against Multi Drug Resistant (MDR) Pathogens?, Applications of Multifunctional Nanomaterials, 2023, Elsevier, 475–498, 10.1016/B978-0-12-820557-0.00008-4.

[43] Jassal M. and Bishai W. R. , Extensively Drug-Resistant Tuberculosis, Lancet Infectious Diseases. (2009) 9, no. 1, 19–30, 10.1016/S1473-3099(08)70260-3, 2-s2.0-57449102564. 18990610

[44] Ghazaei C. , Upcoming Multi-Drug-Resistant and Extensively Drug-Resistant Bacteria, Research in Molecular Medicine. (2022) 10, no. 2, 85–96, 10.32598/rmm.10.2.820.7.

[45] Nasiri M. J. , Rezaei F. , Zamani S. , Darban-Sarokhalil D. , Fooladi A. A. I. , Shojaei H. , and Feizabadi M. M. , Drug Resistance Pattern of *Mycobacterium tuberculosis* Isolates From Patients of Five Provinces of Iran, Asian Pacific Journal of Tropical Medicine. (2014) 7, no. 3, 193–196, 10.1016/S1995-7645(14)60019-5, 2-s2.0-84893514696, 24507638. 24507638

[46] Rabiee N. , Ahmadi S. , Akhavan O. , and Luque R. , Silver and Gold Nanoparticles for Antimicrobial Purposes Against Multi-Drug Resistance Bacteria, Materials. (2022) 15, no. 5, 10.3390/ma15051799, 35269031. PMC8911831 35269031

[47] Van Rie A. , Warren R. , Richardson M. , Gie R. P. , Enarson D. A. , Beyers N. , and Van Helden P. D. , Classification of Drug-Resistant Tuberculosis in an Epidemic Area, Lancet. (2000) 356, no. 9223, 22–25, 10.1016/S0140-6736(00)02429-6, 10892760. 10892760

[48] Fuchs F. , Wille J. , Hamprecht A. , Parcina M. , Lehmann C. , Schwarze-Zander C. , Seifert H. , and Higgins P. G. , In Vitro Activity of Mecillinam and Nitroxoline Against *Neisseria gonorrhoeae*—Re-Purposing Old Antibiotics in the Multi-Drug Resistance Era, Journal of Medical Microbiology. (2019) 68, no. 7, 991–995, 10.1099/jmm.0.001014, 2-s2.0-85069237427, 31162022. 31162022

[49] Chuanchuen R. , Beinlich K. , Hoang T. T. , Becher A. , Karkhoff-Schweizer R. R. , and Schweizer H. P. , Cross-Resistance Between Triclosan and Antibiotics in *Pseudomonas aeruginosa* Is Mediated by Multidrug Efflux Pumps: Exposure of a Susceptible Mutant Strain to Triclosan Selects nfxB Mutants Overexpressing MexCD-OprJ, Antimicrobial Agents and Chemotherapy. (2001) 45, no. 2, 428–432, 10.1128/AAC.45.2.428-432.2001, 2-s2.0-0035139556, 11158736. 11158736 PMC90308

[50] Enemchukwu C. M. , Oli A. N. , Okoye E. I. , Ujam N. T. , Osazuwa E. O. , Emechebe G. O. , Okeke K. N. , Ifezulike C. C. , Ejiofor O. S. , and Okoyeh J. N. , Winning the War Against Multi-Drug Resistant Diarrhoeagenic Bacteria, Microorganisms. (2019) 7, no. 7, 10.3390/microorganisms7070197, 31295889. PMC6680719 31295889

[51] Alekshun M. N. and Levy S. B. , Molecular Mechanisms of Antibacterial Multidrug Resistance, Cell. (2007) 128, no. 6, 1037–1050, 10.1016/j.cell.2007.03.004, 2-s2.0-33947260230. 17382878

[52] Mba I. E. and Nweze E. I. , Antimicrobial Peptides Therapy: An Emerging Alternative for Treating Drug-Resistant Bacteria, Yale Journal of Biology and Medicine. (2022) 95, no. 4, 445–463, 36568838. 36568838 PMC9765339

[53] Sahm D. F. , Mechanisms of Antimicrobial Resistance, Clinical Microbiology Newsletter. (1989) 11, no. 2, 9–14, 10.1016/0196-4399(89)90016-0, 2-s2.0-38249024987.

[54] Page S. W. and Gautier P. , Use of Antimicrobial Agents in Livestock, Revue Scientifique et Technique–OIE. (2012) 31, no. 1, 145–188, 10.20506/rst.31.1.2106, 2-s2.0-84864074521. 22849274

[55] Fair R. J. and Tor Y. , Antibiotics and Bacterial Resistance in the 21st Century, Perspectives in Medicinal Chemistry. (2014) 6, 25–64, 10.4137/PMC.S14459, 2-s2.0-84926642568, 25232278. 25232278 PMC4159373

[56] Meade E. , Slattery M. A. , and Garvey M. , Bacteriocins, Potent Antimicrobial Peptides and the Fight Against Multi Drug Resistant Species: Resistance Is Futile?, Antibiotics. (2020) 9, no. 1, 10.3390/antibiotics9010032, 31963311. PMC7168330 31963311

[57] Gandhi N. R. , Moll A. , Sturm A. W. , Pawinski R. , Govender T. , Lalloo U. , Zeller K. , Andrews J. , and Friedland G. , Extensively Drug-Resistant Tuberculosis as a Cause of Death in Patients Co-Infected With Tuberculosis and HIV in a Rural Area of South Africa, Lancet. (2006) 368, no. 9547, 1575–1580, 10.1016/S0140-6736(06)69573-1, 2-s2.0-33750629139. 17084757

[58] Provenzani A. , Hospodar A. R. , Meyer A. L. , Leonardi Vinci D. , Hwang E. Y. , Butrus C. M. , and Polidori P. , Multidrug-Resistant Gram-Negative Organisms: A Review of Recently Approved Antibiotics and Novel Pipeline Agents, International Journal of Clinical Pharmacy. (2020) 42, no. 4, 1016–1025, 10.1007/s11096-020-01089-y, 32638294. 32638294

[59] Arias C. A. and Murray B. E. , Antibiotic-Resistant Bugs in the 21st Century: A Clinical Super-Challenge, New England Journal of Medicine. (2009) 360, no. 5, 439–443, 10.1056/NEJMp0804651, 2-s2.0-59449088862, 19179312. 19179312

[60] Huang L. , Wu C. , Gao H. , Xu C. , Dai M. , Huang L. , Hao H. , Wang X. , and Cheng G. , Bacterial Multidrug Efflux Pumps at the Frontline of Antimicrobial Resistance: An Overview, Antibiotics. (2022) 11, no. 4, 10.3390/antibiotics11040520, 35453271. PMC9032748 35453271

[61] Nikaido H. , Multidrug Resistance in Bacteria, Annual Review of Biochemistry. (2009) 78, no. 1, 119–146, 10.1146/annurev.biochem.78.082907.145923, 2-s2.0-65249146929, 19231985. PMC2839888 19231985

[62] Vaz-Moreira I. , Nunes O. C. , and Manaia C. M. , Bacterial Diversity and Antibiotic Resistance in Water Habitats: Searching the Links With the Human Microbiome, FEMS Microbiology Reviews. (2014) 38, no. 4, 761–778, 10.1111/1574-6976.12062, 2-s2.0-84903955388, 24484530. 24484530

[63] Mateo E. M. and Jiménez M. , Silver Nanoparticle-Based Therapy: Can It Be Useful to Combat Multi-Drug Resistant Bacteria?, Antibiotics. (2022) 11, no. 9, 10.3390/antibiotics11091205, 36139984. PMC9495113 36139984

[64] National Health Service (NHS) , Antibiotic Resistance, 2017, https://www.nhs.uk/conditions/antibiotics/antibiotic-antimicrobial-resistance/.

[65] Jacoby G. A. and Munoz-Price L. S. , The New *β*-Lactamases, New England Journal of Medicine. (2005) 352, no. 4, 380–391, 10.1056/NEJMra041359, 2-s2.0-19944434187. 15673804

[66] Giedraitienė A. , Vitkauskienė A. , Naginienė R. , and Pavilonis A. , Antibiotic Resistance Mechanisms of Clinically Important Bacteria, Medicina. (2011) 47, no. 3, 10.3390/medicina47030019. 21822035

[67] Hussein M. , Hu X. , Paulin O. K. , Crawford S. , Zhou Q. T. , Baker M. , Schneider-Futschik E. K. , Zhu Y. , Li J. , and Velkov T. , Polymyxin B Combinations With FDA-Approved Non-Antibiotic Phenothiazine Drugs Targeting Multi-Drug Resistance of Gram-Negative Pathogens, Computational and Structural Biotechnology Journal. (2020) 18, 2247–2258, 10.1016/j.csbj.2020.08.008, 32952938. 32952938 PMC7481501

[68] Byarugaba D. K. , Mechanisms of Antimicrobial Resistance, Antimicrobial Resistance in Developing Countries, 2010, Springer New York, New York, NY, 15–26, 10.1007/978-0-387-89370-9_2.

[69] Tang S. S. , Apisarnthanarak A. , and Hsu L. Y. , Mechanisms of *β*-Lactam Antimicrobial Resistance and Epidemiology of Major Community- and Healthcare-Associated Multidrug-Resistant Bacteria, Advanced Drug Delivery Reviews. (2014) 78, 3–13, 10.1016/j.addr.2014.08.003, 2-s2.0-84919491854, 25134490. 25134490

[70] Mancuso G. , Midiri A. , Gerace E. , and Biondo C. , Bacterial Antibiotic Resistance: The Most Critical Pathogens, Pathogens. (2021) 10, no. 10, 10.3390/pathogens10101310, 34684258. PMC8541462 34684258

[71] Santajit S. and Indrawattana N. , Mechanisms of Antimicrobial Resistance in ESKAPE Pathogens, BioMed Research International. (2016) 2016, 2475067, 10.1155/2016/2475067, 2-s2.0-84971342940. 27274985 PMC4871955

[72] Liu Y. , Li R. , Xiao X. , and Wang Z. , Antibiotic Adjuvants: An Alternative Approach to Overcome Multi-Drug Resistant Gram-Negative Bacteria, Critical Reviews in Microbiology. (2019) 45, no. 3, 301–314, 10.1080/1040841X.2019.1599813, 2-s2.0-85070791863, 30985240. 30985240

[73] Brianti E. , Napoli E. , Gaglio G. , Falsone L. , Giannetto S. , Solari Basano F. , Nazzari R. , Latrofa M. S. , Annoscia G. , Tarallo V. D. , Stanneck D. , Dantas-Torres F. , and Otranto D. , Field Evaluation of Two Different Treatment Approaches and Their Ability to Control Fleas and Prevent Canine Leishmaniosis in a Highly Endemic Area, PLoS Neglected Tropical Diseases. (2016) 10, no. 9, e0004987, 10.1371/journal.pntd.0004987, 2-s2.0-84991745964, 27632527. 27632527 PMC5025139

[74] Oliva G. , Roura X. , Crotti A. , Maroli M. , Castagnaro M. , Gradoni L. , Lubas G. , Paltrinieri S. , Zatelli A. , and Zini E. , Guidelines for Treatment of Leishmaniasis in Dogs, Journal of the American Veterinary Medical Association. (2010) 236, no. 11, 1192–1198, 10.2460/javma.236.11.1192, 2-s2.0-77953490995. 20513197

[75] Doliwa C. , Escotte-Binet S. , Aubert D. , Velard F. , Schmid A. , Geers R. , and Villena I. , Induction of Sulfadiazine Resistance In Vitro in *Toxoplasma gondii* , Experimental Parasitology. (2013) 133, no. 2, 131–136, 10.1016/j.exppara.2012.11.019, 2-s2.0-84871400764, 23206954. 23206954

[76] Tonkin M. , Khan S. , Wani M. Y. , and Ahmad A. , Quorum Sensing—A Stratagem for Conquering Multi-Drug Resistant Pathogens, Current Pharmaceutical Design. (2021) 27, no. 25, 2835–2847, 10.2174/1381612826666201210105638, 33302856. 33302856

[77] Elelu N. , Agene G. , Sanusi F. , and Al-Mustapha A. I. , A Cross-Sectional Questionnaire Survey on Knowledge of Anti-Protozoal Drug Use and Resistance Among AHPs in Kwara State, Nigeria, BMC Veterinary Research. (2022) 18, no. 1, 10.1186/s12917-022-03331-3, 35668403. PMC9172141 35668403

[78] Abbas H. , Younus M. , Fareed Z. , Saleem M. M. , Jones M. K. , Raheemi H. , Ijaz A. , and Saleem M. N. , Antiprotozoal Resistance, Antiparasitic Drug Resistance in Veterinary Practice, 2024, CABI, 19–40, 10.1079/9781800622807.0002.

[79] El-Taweel H. A. , Understanding Drug Resistance in Human Intestinal Protozoa, Parasitology Research. (2015) 114, no. 5, 1647–1659, 10.1007/s00436-015-4423-1, 2-s2.0-84940006264, 25782683. 25782683

[80] Nweze J. A. , Mbaoji F. N. , Huang G. , Li Y. , Yang L. , Zhang Y. , Huang S. , Pan L. , and Yang D. , Antibiotics Development and the Potentials of Marine-Derived Compounds to Stem the Tide of Multidrug-Resistant Pathogenic Bacteria, Fungi, and Protozoa, Marine Drugs. (2020) 18, no. 3, 10.3390/md18030145, 32121196. PMC7142797 32121196

[81] Pramanik P. K. , Alam M. N. , Chowdhury D. R. , and Chakraborti T. , Drug Resistance in Protozoan Parasites: An Incessant Wrestle for Survival, Journal of Global Antimicrobial Resistance. (2019) 18, 1–11, 10.1016/j.jgar.2019.01.023, 2-s2.0-85066624787, 30685461. 30685461

[82] Borst P. and Ouellette M. , New Mechanisms of Drug Resistance in Parasitic Protozoa, Annual Review of Microbiology. (1995) 49, no. 1, 427–460, 10.1146/annurev.mi.49.100195.002235, 2-s2.0-0028822932. 8561467

[83] Upcroft P. and Upcroft J. A. , Drug Targets and Mechanisms of Resistance in the Anaerobic Protozoa, Clinical Microbiology Reviews. (2001) 14, no. 1, 150–164, 10.1128/CMR.14.1.150-164.2001, 2-s2.0-0035138734, 11148007. 11148007 PMC88967

[84] Klokouzas A. , Shahi S. , Hladky S. B. , Barrand M. A. , and van Veen H. W. , ABC Transporters and Drug Resistance in Parasitic Protozoa, International Journal of Antimicrobial Agents. (2003) 22, no. 3, 301–317, 10.1016/S0924-8579(03)00210-3, 2-s2.0-0042764352. 13678838

[85] Pradines B. , ABC Proteins Involved in Protozoan Parasite Resistance, ABC Transporters and Multidrug Resistance, 2009, 195–238, 10.1002/9780470495131.ch7, 2-s2.0-77953827737.

[86] Andrews K. T. , Fisher G. , and Skinner-Adams T. S. , Drug Repurposing and Human Parasitic Protozoan Diseases, International Journal for Parasitology: Drugs and Drug Resistance. (2014) 4, no. 2, 95–111, 10.1016/j.ijpddr.2014.02.002, 2-s2.0-84897973164, 25057459. 25057459 PMC4095053

[87] Karpe A. V. , Beale D. J. , and Tran C. D. , Intelligent Biological Networks: Improving Antimicrobial Resistance Resilience Through Nutritional Interventions to Understand Protozoal Gut Infections, Microorganisms. (2023) 11, no. 7, 10.3390/microorganisms11071800, 37512972. PMC10383877 37512972

[88] Kaplan R. M. , Drug Resistance in Nematodes of Veterinary Importance: A Status Report, Trends in Parasitology. (2004) 20, no. 10, 477–481, 10.1016/j.pt.2004.08.001, 2-s2.0-4444375921, 15363441. 15363441

[89] Hotez P. J. , Brindley P. J. , Bethony J. M. , King C. H. , Pearce E. J. , and Jacobson J. , Helminth Infections: The Great Neglected Tropical Diseases, Journal of Clinical Investigation. (2008) 118, no. 4, 1311–1321, 10.1172/JCI34261, 2-s2.0-41849114954, 18382743. 18382743 PMC2276811

[90] Papaneophytou C. , Giannenas I. , and Dragomir C. , Resistance of Bacteria, Fungi, and Parasites to Antibiotics or Natural Substances of Botanical Origin, Feed Additives, 2020, Elsevier, 339–354, 10.1016/B978-0-12-814700-9.00019-4.

[91] Gilleard J. S. , Understanding Anthelmintic Resistance: The Need for Genomics and Genetics, International Journal for Parasitology. (2006) 36, no. 12, 1227–1239, 10.1016/j.ijpara.2006.06.010, 2-s2.0-33748459194, 16889782. 16889782

[92] Voisey J. and Morris C. P. , SNP Technologies for Drug Discovery: A Current Review, Current Drug Discovery Technologies. (2008) 5, no. 3, 230–235, 10.2174/157016308785739811, 2-s2.0-51349132793, 18690891. 18690891

[93] Zhou S. F. , Structure, Function and Regulation of P-Glycoprotein and Its Clinical Relevance in Drug Disposition, Xenobiotica. (2008) 38, no. 7–8, 802–832, 10.1080/00498250701867889, 2-s2.0-47349110179, 18668431. 18668431

[94] Malnoë D. , Fardel O. , and Le Corre P. , Involvement of Transporters in Intestinal Drug–Drug Interactions of Oral Targeted Anticancer Drugs Assessed by Changes in Drug Absorption Time, Pharmaceutics. (2022) 14, no. 11, 10.3390/pharmaceutics14112493, 36432682. PMC9696196 36432682

[95] Ouellette M. and Ward S. A. , Drug Resistance in Parasites, Molecular Medical Parasitology, 2003, Elsevier, 397–432, 10.1016/B978-012473346-6/50019-3.

[96] Jones P. M. and George A. M. , Multidrug Resistance in Parasites: ABC Transporters, P-Glycoproteins and Molecular Modelling, International Journal for Parasitology. (2005) 35, no. 5, 555–566, 10.1016/j.ijpara.2005.01.012, 2-s2.0-17144426125, 15826647. 15826647

[97] El-Bassiouni E. A. , Helmy M. H. , Saad E. I. , Kamel E. N. , Abdel-Meguid E. , and Hussein H. S. E. , Modulation of the Antioxidant Defence in Different Developmental Stages of *Schistosoma mansoni* by Praziquantel and Artemether, British Journal of Biomedical Science. (2007) 64, no. 4, 168–174, 10.1080/09674845.2007.11732782, 2-s2.0-37848999455, 18236739. 18236739

[98] Singh S. , Dwivedi N. , Gupta M. , Dwivedi A. , Prakash J. , and Gupta V. , Antimicrobial Resistance and Recent Advancement to Combat Parasitic Infections; Development of Resistance to Antihelminthic/Antiprotozoal and Antimalarial Drugs, Advances in Antiparasitic Therapies and Drug Delivery, 2024, Academic Press, 289–332, 10.1016/B978-0-443-15178-1.00006-7.

[99] Strasfeld L. and Chou S. , Antiviral Drug Resistance: Mechanisms and Clinical Implications, Infectious Disease Clinics. (2010) 24, no. 3, 809–833, 10.1016/j.idc.2010.07.001, 2-s2.0-77955251899, 20674805. 20674805

[100] McKeegan K. S. , Borges-Walmsley M. I. , and Walmsley A. R. , Microbial and Viral Drug Resistance Mechanisms, Trends in Microbiology. (2002) 10, no. 10, s8–s14, 10.1016/S0966-842X(02)02429-0, 2-s2.0-0036791919. 12377562

[101] Saidijam M. , Benedetti G. , Ren Q. , Xu Z. , Hoyle C. J. , Palmer S. L. , Ward A. , Bettaney K. E. , Szakonyi G. , Meuller J. , and Morrison S. , Microbial Drug Efflux Proteins of the Major Facilitator Superfamily, Current Drug Targets. (2006) 7, no. 7, 793–811, 10.2174/138945006777709575, 2-s2.0-33745741591. 16842212

[102] Omrani A. S. and Pillay D. , Multi-Drug Resistant HIV-1, Journal of Infection. (2000) 41, no. 1, 5–11, 10.1053/jinf.2000.0702, 2-s2.0-0033836451. 10942635

[103] Iszatt J. J. , Larcombe A. N. , Chan H. K. , Stick S. M. , Garratt L. W. , and Kicic A. , Phage Therapy for Multi-Drug Resistant Respiratory Tract Infections, Viruses. (2021) 13, no. 9, 10.3390/v13091809, 34578390. PMC8472870 34578390

[104] Loeffler J. and Stevens D. A. , Antifungal Drug Resistance, Clinical Infectious Diseases. (2003) 36, no. supplement 1, S31–S41, 10.1086/344658, 2-s2.0-0037439383. 12516028

[105] Sekyere J. O. and Asante J. , Emerging Mechanisms of Antimicrobial Resistance in Bacteria and Fungi: Advances in the Era of Genomics, Future Microbiology. (2018) 13, no. 2, 241–262, 10.2217/fmb-2017-0172, 2-s2.0-85048897892, 29319341. 29319341

[106] Pérez-Rodríguez F. and Mercanoglu Taban B. , A State-of-Art Review on Multi-Drug Resistant Pathogens in Foods of Animal Origin: Risk Factors and Mitigation Strategies, Frontiers in Microbiology. (2019) 10, 10.3389/fmicb.2019.02091, 2-s2.0-85072826043, 31555256. PMC6742700 31555256

[107] Svanborg C. , Urinary Tract Infections in Children: Microbial Virulence Versus Host Susceptibility, Hot Topics in Infection and Immunity in Children IX, 2013, Springer New York, New York, NY, 205–210, 10.1007/978-1-4614-4726-9_17, 23654069. 23654069

[108] Hendrickson J. A. , Hu C. , Aitken S. L. , and Beyda N. , Antifungal Resistance: A Concerning Trend for the Present and Future, Current Infectious Disease Reports. (2019) 21, no. 12, 1–8, 10.1007/s11908-019-0702-9. 31734730

[109] Mosquera J. , Antifungal Drug Resistance in Aspergillus, 2002, PhD thesis, University of Manchester.

[110] Engle K. and Kumar G. , Tackling Multi-Drug Resistant Fungi by Efflux Pump Inhibitors, Biochemical Pharmacology. (2024) 226, 116400, 10.1016/j.bcp.2024.116400, 38945275. 38945275

[111] Denning D. W. , Antifungal Drug Resistance: An Update, European Journal of Hospital Pharmacy. (2022) 29, no. 2, 109–112, 10.1136/ejhpharm-2020-002604, 35190454. 35190454 PMC8899664

[112] Latgé J. P. , *Aspergillus* fumigatusand Aspergillosis, Clinical Microbiology Reviews. (1999) 12, no. 2, 310–350, 10.1128/CMR.12.2.310. 10194462 PMC88920

[113] Arikan-Akdagli S. , Ghannoum M. , and Meis J. F. , Antifungal Resistance: Specific Focus on Multidrug Resistance in *Candida auris* and Secondary Azole Resistance in *Aspergillus fumigatus* , Journal of Fungi. (2018) 4, no. 4, 10.3390/jof4040129, 2-s2.0-85058019162, 30563053. PMC6308933 30563053

[114] Roe K. , Treatment Alternatives for Multidrug-Resistant Fungal Pathogens, Drug Discovery Today. (2023) 28, no. 6, 103596, 10.1016/j.drudis.2023.103596. 37086779

[115] Howden B. P. , Slavin M. A. , Schwarer A. P. , and Mijch A. M. , Successful Control of Disseminated *Scedosporium prolificans* Infection With a Combination of Voriconazole and Terbinafine, European Journal of Clinical Microbiology and Infectious Diseases. (2003) 22, no. 2, 111–113, 10.1007/s10096-002-0877-z, 12627286. 12627286

[116] Gulshan K. and Moye-Rowley W. S. , Multidrug Resistance in Fungi, Eukaryotic Cell. (2007) 6, no. 11, 1933–1942, 10.1128/EC.00254-07, 2-s2.0-36849043112, 17873085. 17873085 PMC2168405

[117] Khan R. , Islam B. , Akram M. , Shakil S. , Ahmad A. , Ali S. M. , Siddiqui M. , and Khan A. U. , Antimicrobial Activity of Five Herbal Extracts Against Multi Drug Resistant (MDR) Strains of Bacteria and Fungus of Clinical Origin, Molecules. (2009) 14, no. 2, 586–597, 10.3390/molecules14020586, 2-s2.0-61449203801, 19214149. 19214149 PMC6253777

[118] Perlin D. S. , Rautemaa-Richardson R. , and Alastruey-Izquierdo A. , The Global Problem of Antifungal Resistance: Prevalence, Mechanisms, and Management, Lancet Infectious Diseases. (2017) 17, no. 12, e383–e392, 10.1016/S1473-3099(17)30316-X, 2-s2.0-85026531535, 28774698. 28774698

[119] Osset-Trénor P. , Pascual-Ahuir A. , and Proft M. , Fungal Drug Response and Antimicrobial Resistance, Journal of Fungi. (2023) 9, no. 5, 10.3390/jof9050565, 37233275. PMC10219139 37233275

[120] Paterson D. L. and Bonomo R. A. , Extended-Spectrum *β*-Lactamases: A Clinical Update, Clinical Microbiology Reviews. (2005) 18, no. 4, 657–686, 10.1128/CMR.18.4.657-686.2005, 2-s2.0-27144490073, 16223952. 16223952 PMC1265908

[121] Deurenberg R. H. , Vink C. , Kalenic S. , Friedrich A. W. , Bruggeman C. A. , and Stobberingh E. E. , The Molecular Evolution of Methicillin-Resistant *Staphylococcus aureus* , Clinical Microbiology and Infection. (2007) 13, no. 3, 222–235, 10.1111/j.1469-0691.2006.01573.x, 2-s2.0-34247215693, 17391376. 17391376

[122] Muhammad R. H. , Yaro C. A. , Balarabe M. B. , Zainab J. A. , and Adedayo M. R. , Assessment of Bacteria Associated with Ready-to-Eat Food Sold at Federal University Dutse, Jigawa State, Nigeria, International Journal of Current Research in Biosciences and Plant Biology. (2016) 3, no. 4, 5–14, 10.20546/ijcrbp.2016.304.002.

[123] Pontes J. T. C. D. , Toledo Borges A. B. , Roque-Borda C. A. , and Pavan F. R. , Antimicrobial Peptides as an Alternative for the Eradication of Bacterial Biofilms of Multi-Drug Resistant Bacteria, Pharmaceutics. (2022) 14, no. 3, 10.3390/pharmaceutics14030642, 35336016. PMC8950055 35336016

[124] Centers for Disease Control and Prevention (CDC) , Vancomycin-Resistant Enterococci, 2004, MDPI, Basel, Switzerland, https://wwwnc.cdc.gov/eid/article/11/6/04-1204.

[125] Palma E. , Tilocca B. , and Roncada P. , Antimicrobial Resistance in Veterinary Medicine: An Overview, International Journal of Molecular Sciences. (2020) 21, no. 6, 10.3390/ijms21061914, 32168903. PMC7139321 32168903

[126] Paterson D. L. and Bonomo R. A. , Multidrug-Resistant Gram-Negative Pathogens: The Urgent Need for ‘Old’ Polymyxins, Polymyxin Antibiotics: From Laboratory Bench to Bedside, 2019, Springer International Publishing, Cham, 9–13, 10.1007/978-3-030-16373-0_2, 2-s2.0-85070533994, 31364068. 31364068

[127] Ude Z. , Flothkötter N. , Sheehan G. , Brennan M. , Kavanagh K. , and Marmion C. J. , Multi-Targeted Metallo-Ciprofloxacin Derivatives Rationally Designed and Developed to Overcome Antimicrobial Resistance, International Journal of Antimicrobial Agents. (2021) 58, no. 6, 106449, 10.1016/j.ijantimicag.2021.106449, 34644603. 34644603

[128] Martinez J. L. , Baquero F. , and Andersson D. I. , Beyond Serial Passages: New Methods for Predicting the Emergence of Resistance to Novel Antibiotics, Current Opinion in Pharmacology. (2011) 11, no. 5, 439–445, 10.1016/j.coph.2011.07.005, 2-s2.0-80053124273, 21835695. 21835695

[129] Urban-Chmiel R. , Marek A. , Stępień-Pyśniak D. , Wieczorek K. , Dec M. , Nowaczek A. , and Osek J. , Antibiotic Resistance in Bacteria—A Review, Antibiotics. (2022) 11, no. 8, 10.3390/antibiotics11081079, 36009947. PMC9404765 36009947

[130] Munita J. M. and Arias C. A. , Mechanisms of Antibiotic Resistance, Virulence Mechanisms of Bacterial Pathogens, 2016, 4th edition, ASM Press, Washington, DC, USA, 481–511, 10.1128/9781555819286.ch17.

[131] Parveen M. and Some S. , Antimicrobial Activity of Medicinal Plants: A Weapon to Combat Multi-Drug Resistant Pathogens, International Journal of Plant and Environment. (2021) 7, no. 2, 133–141, 10.18811/ijpen.v7i02.03.

[132] Yanat B. , Rodríguez-Martínez J. M. , and Touati A. , Plasmid-Mediated Quinolone Resistance in Enterobacteriaceae: A Systematic Review With a Focus on Mediterranean Countries, European Journal of Clinical Microbiology & Infectious Diseases. (2017) 36, no. 3, 421–435, 10.1007/s10096-016-2847-x, 2-s2.0-84997246198, 27889879. 27889879

[133] Vivas R. , Barbosa A. A. T. , Dolabela S. S. , and Jain S. , Multidrug-Resistant Bacteria and Alternative Methods to Control Them: An Overview, Microbial Drug Resistance. (2019) 25, no. 6, 890–908, 10.1089/mdr.2018.0319, 2-s2.0-85064835800, 30811275. 30811275

[134] Food and Drug Administration (FDA) , General Principles for the Development of New Antibiotics, 2004, Mary Ann Liebert,, Larchmont, N.Y., https://www.fda.gov/.

[135] Delcour A. H. , Outer Membrane Permeability and Antibiotic Resistance, Biochimica et Biophysica Acta (BBA)-Proteins and Proteomics. (2009) 1794, no. 5, 808–816, 10.1016/j.bbapap.2008.11.005, 2-s2.0-64649088018, 19100346. 19100346 PMC2696358

[136] Valenti G. E. , Alfei S. , Caviglia D. , Domenicotti C. , and Marengo B. , Antimicrobial Peptides and Cationic Nanoparticles: A Broad-Spectrum Weapon to Fight Multi-Drug Resistance Not Only in Bacteria, International Journal of Molecular Sciences. (2022) 23, no. 11, 10.3390/ijms23116108, 35682787. PMC9181033 35682787

[137] Wahab S. , Khan T. , Adil M. , and Khan A. , Mechanistic Aspects of Plant-Based Silver Nanoparticles Against Multi-Drug Resistant Bacteria, Heliyon. (2021) 7, no. 7, e07565, 10.1016/j.heliyon.2021.e07448. 34286126 PMC8273360

[138] Bush K. , The ABCD′s of *β*-Lactamase Nomenclature, Journal of Infection and Chemotherapy. (2013) 19, no. 4, 549–559, 10.1007/s10156-013-0640-7, 2-s2.0-84882239083, 23828655. 23828655

[139] Paulsen I. T. , Multidrug Efflux Pumps and Resistance: Regulation and Evolution, Current Opinion in Microbiology. (2003) 6, no. 5, 446–451, 10.1016/j.mib.2003.08.005, 2-s2.0-0242291983. 14572535

[140] Piddock L. J. , Multidrug-Resistance Efflux Pumps—Not Just for Resistance, Nature Reviews Microbiology. (2006) 4, no. 8, 629–636, 10.1038/nrmicro1464, 2-s2.0-33747154459, 16845433. 16845433

[141] Floyd J. T. , Kumar S. , Mukherjee M. M. , He G. , and Varela M. F. , A Review of the Molecular Mechanisms of Drug Efflux in Pathogenic Bacteria: A Structure-Function Perspective, Recent Research Developments in Membrane Biology. (2013) 3, 15–66.

[142] Poole K. , Efflux-Mediated Antimicrobial Resistance, Journal of Antimicrobial Chemotherapy. (2005) 56, no. 1, 20–51, 10.1093/jac/dki171, 2-s2.0-24044514016. 15914491

[143] Zhang F. and Cheng W. , The Mechanism of Bacterial Resistance and Potential Bacteriostatic Strategies, Antibiotics. (2022) 11, no. 9, 10.3390/antibiotics11091215, 36139994. PMC9495013 36139994

[144] Mulat M. , Pandita A. , and Khan F. , Medicinal Plant Compounds for Combating the Multi-Drug Resistant Pathogenic Bacteria: A Review, Current Pharmaceutical Biotechnology. (2019) 20, no. 3, 183–196, 10.2174/1872210513666190308133429, 2-s2.0-85067279072, 30854956. 30854956

[145] Murugaiyan J. , Kumar P. A. , Rao G. S. , Iskandar K. , Hawser S. , Hays J. P. , Mohsen Y. , Adukkadukkam S. , Awuah W. A. , Jose R. A. M. , Sylvia N. , Nansubuga E. P. , Tilocca B. , Roncada P. , Roson-Calero N. , Moreno-Morales J. , Amin R. , Kumar B. K. , Kumar A. , Toufik A. R. , Zaw T. N. , Akinwotu O. O. , Satyaseela M. P. , and van Dongen M. B. M. , Progress in Alternative Strategies to Combat Antimicrobial Resistance: Focus on Antibiotics, Antibiotics. (2022) 11, no. 2, 10.3390/antibiotics11020200, 35203804. PMC8868457 35203804

[146] Richard Y. , Resistance to Antibiotics and Public Health, 1986, CABI Digital Library.

[147] Okeke I. N. , Laxminarayan R. , Bhutta Z. A. , Duse A. G. , Jenkins P. , O’Brien T. F. , Pablos-Mendez A. , and Klugman K. P. , Antimicrobial Resistance in Developing Countries. Part I: Recent Trends and Current Status, Lancet Infectious Diseases. (2005) 5, no. 8, 481–493, 10.1016/S1473-3099(05)70189-4, 2-s2.0-22544447420, 16048717. 16048717

[148] Miryala S. K. and Ramaiah S. , Exploring the Multi-Drug Resistance in *Escherichia coli* O157:H7 by Gene Interaction Network: A Systems Biology Approach, Genomics. (2019) 111, no. 4, 958–965, 10.1016/j.ygeno.2018.06.002, 2-s2.0-85048790179, 29908320. 29908320

[149] Hermans P. W. , Sluijter M. , Elzenaar K. , van Veen A. , Schonkeren J. J. , Nooren F. M. , van Leeuwen W. J. , de Neeling A. J. , van Klingeren B. , Verbrugh H. A. , and de Groot R. , Penicillin-Resistant *Streptococcus pneumoniae* in the Netherlands: Results of a 1-Year Molecular Epidemiologic Survey, Journal of Infectious Diseases. (1997) 175, no. 6, 1413–1422, 10.1086/516474, 2-s2.0-16944362655, 9180181. 9180181

[150] Szczepanowski R. , Linke B. , Krahn I. , Gartemann K. H. , Gutzkow T. , Eichler W. , Puhler A. , and Schluter A. , Detection of 140 Clinically Relevant Antibiotic-Resistance Genes in the Plasmid Metagenome of Wastewater Treatment Plant Bacteria Showing Reduced Susceptibility to Selected Antibiotics, Microbiology. (2009) 155, no. 7, 2306–2319, 10.1099/mic.0.028233-0, 2-s2.0-69949162470, 19389756. 19389756

[151] Mishra A. , Pradhan D. , Halder J. , Biswasroy P. , Rai V. K. , Dubey D. , Kar B. , Ghosh G. , and Rath G. , Metal Nanoparticles Against Multi-Drug-Resistance Bacteria, Journal of Inorganic Biochemistry. (2022) 237, 111938, 10.1016/j.jinorgbio.2022.111938. 36122430

[152] Woodhead M. , Blasi F. , Ewig S. , Huchon G. , Leven M. , Ortqvist A. , Schaberg T. , Torres A. , van der Heijden G. , and Veheij T. J. M. , Guidelines for the Management of Adult Lower Respiratory Tract Infections, European Respiratory Journal. (2005) 26, no. 6, 1138–1180, 10.1183/09031936.05.00055705, 2-s2.0-29244469121. 16319346

[153] Mohamed D. S. , Abd El-Baky R. M. , Sandle T. , Mandour S. A. , and Ahmed E. F. , Antimicrobial Activity of Silver-Treated Bacteria Against Other Multi-Drug Resistant Pathogens in Their Environment, Antibiotics. (2020) 9, no. 4, 10.3390/antibiotics9040181, 32326384. PMC7235873 32326384

[154] Peterson E. and Kaur P. , Antibiotic Resistance Mechanisms in Bacteria: Relationships Between Resistance Determinants of Antibiotic Producers, Environmental Bacteria, and Clinical Pathogens, Frontiers in Microbiology. (2018) 9, 10.3389/fmicb.2018.02928, 2-s2.0-85057608761, 30555448. PMC6283892 30555448

[155] Mukherjee R. , Priyadarshini A. , Pandey R. P. , and Raj V. S. , Antimicrobial Resistance *inStaphylococcus aureus* , Insights into Drug Resistance in Staphylococcus aureus, 2021, IntechOpen, 11–20, 10.5772/intechopen.96888.

[156] Morris S. and Cerceo E. , Trends, Epidemiology, and Management of Multi-Drug Resistant Gram-Negative Bacterial Infections in the Hospitalized Setting, Antibiotics. (2020) 9, no. 4, 10.3390/antibiotics9040196, 32326058. PMC7235729 32326058

